# SARS‐CoV‐2 Antibody Levels and Infections in Multiple Vaccinated Employees Over Time

**DOI:** 10.1002/jmv.70628

**Published:** 2025-10-18

**Authors:** Ingrid Sander, Sabine Kespohl, Alexandra Beine, Kerstin Belting, Jürgen Bünger, Simon Weidhaas, Ingolf Hosbach, Christian Eisenhawer, Jan Gleichenhagen, Philipp Göcke, Thomas Brüning, Monika Raulf

**Affiliations:** ^1^ Institute for Prevention and Occupational Medicine of the German Social Accident Insurance Institute of the Ruhr University Bochum (IPA) Bochum Germany; ^2^ Practice for Laboratory Medicine and Microbiology Bochum Bochum Germany

**Keywords:** antibody levels, half‐life of specific IgG, nucleocapsid, Omicron, SARS‐CoV‐2 vaccination, spike

## Abstract

This prospective cohort study aimed to monitor the humoral immune response to SARS‐CoV‐2 proteins over 3 years in relation to repeated vaccination or infection. Quantitative immunoassays traceable to international IgG units were used to measure and compare IgG levels against Wuhan type and Omicron spike‐S1 and nucleocapsid proteins over 3 years. In addition, the Euroimmun‐N‐test was used to determine positive IgG values against the nucleocapsid. A total of 1223 serum samples from 126 participants without evidence of COVID‐19 at enrollment were analyzed and used to calculate antibody half‐lives following vaccination or infection, as reported by the questionnaire. Antibody levels and half‐life against both variants of the spike S1 protein increased significantly with each additional vaccination or after infection. IgG against the Wuhan type was, on average, three times higher than against the Omicron variant. The half‐lives of antibodies against the Wuhan type after the second and third vaccinations and after infection were significantly longer than those of IgG against the Omicron variant. Two‐thirds of the cohort reported COVID‐19 infection, detected with a sensitivity of 70% by the quantitative nucleocapsid IgG assay and 83% by the Euroimmun‐N‐test. The level of anti‐nucleocapsid‐IgG after infection in the vaccinated cohort was significantly lower than anti‐S1‐IgG and also lower than in unvaccinated infected persons. Despite repeated vaccinations and progressively increasing IgG antibody levels targeting the spike protein, most of the cohort reported breakthrough infections, possibly due to the lower concentration and reduced half‐life of antibodies against the Omicron variant.

## 1 Introduction

The coronavirus, SARS‐CoV‐2, leading to the illness known as COVID‐19, caused a global health crisis that was declared a pandemic by the World Health Organization (WHO) on March 11, 2020 [1]. Since the first COVID‐19 vaccines were licensed in late 2020, they have played a crucial role in the global effort to control the pandemic by providing immunity and reducing the severity of the disease [2]. The first licensed vaccines deliver genetic sequences of the spike‐S1 protein into human cells. The Pfizer‐BioNTech and Moderna vaccines do this via messenger RNA (mRNA) [3], while the AstraZeneca‐Oxford vaccine AZD1222 uses a modified adenovirus [4]. The duration of immunity, whether from natural infection or vaccination, is a key measure of protection. In addition to cellular immunity, virus‐specific antibodies play an important role in this protection. Initially, COVID‐19 vaccination strategies focused on administering two doses of the above‐mentioned vaccines to generate strong immunity against SARS‐CoV‐2. However, as the pandemic progressed, it became clear that the immunity conferred by these initial doses was waning over time, particularly with the emergence of new variants. Subsequent booster doses were introduced to enhance the immune response and provide continued protection against emerging variants [5]. To monitor the humoral immune response after infection or vaccination, we have developed quantitative enzyme linked immuno sorbent assays (ELISAs) to measure the concentration of IgG to the Wuhan type spike‐S1 protein produced in human embryonic kidney 293 cells and to the nucleocapsid protein produced in *E. coli* cells [6]. Both ELISAs gave repeatable results traceable to international units because of their parallelism to WHO reference preparations. The sera of a study group of 144 COVID‐19 positive subjects infected before vaccination had median IgG concentrations of 4 mg_A_ L^−1^ (equivalent to 102 “Binding activity units” BAU mL^−1^) against the Wuhan type spike‐S1 protein and 13 mg_A_ L^−1^ (equivalent to 84 BAU mL^−1^) against the nucleocapsid protein [6].

Especially after the Omicron variant of SARS‐CoV‐2 became dominant in early 2022, numerous breakthrough infections occurred [7, 8]. We therefore developed an additional quantitative IgG ELISA targeting this variant of the spike‐S1 protein, which differs from the wild‐type variant by 30 mutations.

The aim of our longitudinal cohort study, conducted over a period of 3 years from the start of the vaccination campaign in 2021, was to monitor SARS‐CoV‐2 IgG antibody levels over time in relation to repeated vaccination or infection. For this purpose, up to 18 blood samples per person were collected during the study from a cohort of 126 people who had no evidence of COVID‐19 at enrollment. Each sample was accompanied by a questionnaire to document evidence of COVID‐19 by PCR or antigen testing. Understanding the sustainability of the immune response after vaccination and before and after infection is crucial for medical decision‐making and further vaccination strategies. Analysis of the concentration and half‐life of IgG antibodies to different SARS‐CoV‐2 proteins and variants should help to establish objective criteria for defining the degree and duration of protective immunity at the individual level.

## 2 Methods

### 2.1 Study Participants

The study was conducted in accordance with the World Medical Association's Code of Ethics for Human Research (Declaration of Helsinki) and was approved by the local ethics committee of the Ruhr University Bochum in Germany (registration no. 20‐7007, 2020‐09‐04 and amendment, 2021‐02‐01). The study started in February 2021, after vaccines became available in Germany, and ended 3 years later. Employees of our research institute and, in individual cases, relatives or friends, participated voluntarily in the study after informed consent and donated a blood sample before and after vaccination or, optionally, after detection of infection by PCR or antigen test. For each serum sample, a one‐page questionnaire with a pseudonymized study number was filled in, indicating dates and vaccines as well as PCR or rapid antigen test results for SARS‐CoV‐2 infection. In addition to free rapid antigen tests for citizens in Germany (March 2021 to June 2022), the prevention concept at our institute included regular rapid antigen tests before starting work on site.

Blood samples were collected before the first vaccination, between 14 and 21 days after the first vaccination, but before the second vaccination, and approximately 60, 90, 120, 240, 360, 480, and 720 days after the first vaccination. In addition, blood samples could be taken about 14 days after a further vaccination or infection. Since November 2022, the questionnaire has been updated with additional vaccine variants and a page asking whether symptoms of the disease occurred shortly before or after evidence of infection by positive PCR or antigen tests. A total of 1223 serum samples from 126 participants were included in the study and analyzed.

The following vaccines were used by participants of the study: mRNA BNT162b2, BNT BA.4‐5, BNT BA.1 (Comirnaty, BioNTech, Mainz, Germany), mRNA‐1273, mRNA BA.4‐5 (Moderna Cambridge, MA, USA), and adenovirus‐vectored ChAdOx1 nCoV‐19 (AZD1222, Vaczevria, AstraZeneca, Cambridge, UK).

### 2.2 SARS‐CoV‐2 ELISAs

Before vaccines against the SARS‐CoV‐2 virus were licensed, quantitative IgG ELISAs were developed in our laboratory and standardized against internationally recognized reference preparations. Both the recombinant SARS‐CoV‐2 S1 protein and the nucleocapsid protein, based on the sequence of the Wuhan virus, were coated onto microtiter plates, and the binding of human IgG antibodies was quantified using a biotinylated pan‐anti‐human‐FC‐IgG monoclonal antibody. The details of the components and the reference curve procedure for quantifying specific IgG concentrations in mg_A_ L^−1^, including standardization and validation of the method, are described in 2022 [6]. For the S1 ELISA (IPA S1 test), all IgG values above the detection limit (0.5 mg_A_ L^−1^) of the ELISA were considered positive. For the nucleocapsid ELISA (IPA N test), concentrations below 3.1 mg_A_ L^−1^ were considered negative, between 3.1 and 5 mg_A_ L^−1^ as borderline and concentrations ≥ 5 mg_A_ L^−1^ as positive.

In addition, we established an ELISA to measure specific IgG to the Omicron variant of the S1 protein (IPA oS1 test). Therefore, B.1.1.529/Omicron SARS‐CoV‐2 spike‐S1 with a poly‐histidine tag at the C‐terminus, expressed in human embryonic kidney 293 (HEK293) cells, was obtained from Acro Biosystems (Switzerland, Basel, Cat No. S1N‐C52Ha), and coated onto microtiter plates. The final coating conditions (250 ng per well in 0.1 M carbonate‐bicarbonate buffer pH 9.6) and all other assay components were the same as those used for the original recombinant SARS‐CoV‐2 S1 protein (Sino Biological, Ashburn, Germany, 40591‐V08H), described in 2022 [6]. When the WHO reference standard 20/136, derived from pooled human plasma of convalescent COVID‐19 patients and containing 1000 binding activity units per milliliter (BAU mL^−1^) [9], was tested in the anti‐oS1 assay, an IgG concentration of 10.7 mg_A_ L^−1^ was measured. In comparison, this reference standard was measured in the IPA S1 assay to yield 40 mg_A_ L^−1^ anti‐S1‐IgG and in the IPA N assay to yield 150 mg_A_ L^−1^ anti‐N‐IgG [6].

In addition, all sera were analyzed using the following commercial anti‐SARS‐CoV‐2 IgG immunoassay which was used according to the manufacturers' instructions: anti‐nucleocapsid IgG ELISA (Euroimmun N test, Anti‐SARS‐CoV‐2‐NCP, EI 2606‐9620‐2G, Euroimmun, Lübeck, Germany). The manufacturer's cut‐off values were used: Ratios between 0.8 and 1.1 were considered borderline, and values ≥ 1.1 were considered positive.

### 2.3 Statistical Analysis

Questionnaire data and IgG ELISA results were entered into a Microsoft Access database and analyzed using MS Excel (Microsoft, Office 365) and GraphPad Prism 10.3.1 (GraphPad Software, San Diego, California).

The following definitions and formulae were used to calculate the half‐life (t_1/2_) of sIgG in days after the 2nd, 3rd, or 4th vaccination or after infection:

c0: initial concentration of sIgG after event 1

c(t): final concentration of sIgG at time t before event 2

t: time between sampling dates of the initial and final concentration

k: decay constant

$$k=\frac{1}{t}*\ln\left(\frac{c0}{c\left(t\right)}\right)$$

$$t\;1/2=\frac{\ln\left(2\right)}{k}$$

For anti‐S1‐IgG, vaccination and infection are considered events, and for anti‐nucleocapsid IgG, only infection is considered an event. When IgG values were present at more than two time points between two events, all data pairs were used to calculate the decay constant *k*, and then the mean of *k* values > 0 were used to calculate t_1/2_. The decay constant of sIgG to the nucleocapsid was only calculated if the c0 value was > 3.1 mg_A_ L^−1^ after infection.

Specific IgG and half‐life values were tested for normality or log‐normality and then analyzed by Spearman's correlation, Kruskal−Wallis test, and Dunn's test (unpaired due to missing samples at some time points) or by Wilcoxon matched‐pairs signed‐rank test or Mann−Whitney *U* test to investigate influences of vaccination, vaccine, or sex. In addition, sIgG and half‐life values were log‐transformed and their linear regression against age was calculated. The null hypothesis of slope = 0 was then tested by the extra sum‐of‐squares *F* test. If the null hypothesis was rejected, the function of the fitted line was calculated.

## 3 Results

### 3.1 Concordance of Anti‐Nucleocapsid IgG Results

One hundred and twenty‐six participants aged between 18 and 67 years were included in the analysis, with 2−18 serum samples collected. All enrolled participants had no evidence of SARS‐CoV‐2 infection before vaccination and were vaccinated at least once during the study (Table 1). Evidence of SARS‐CoV‐2 infection based on positive PCR or rapid antigen tests was recorded using the questionnaire. In addition, antibodies to SARS‐CoV‐2 nucleocapsid are indicative of infection, and the concordance between IPA‐N‐test and Euroimmun‐N‐test results in relation to questionnaire‐reported infections is shown in Table 2. Of 65 specimens collected 10 to 100 days after a positive PCR or antigen test was reported, 51 (78%) had a positive Euroimmun‐N‐test result and 34 (52%) showed a positive IPA N‐test result, with 32 (49%) being positive in both tests (Table 2a). Even when borderline results are considered positive, the sensitivity of the nucleocapsid antibody result as an indication of a SARS‐CoV‐2 infection was only 83% (Euroimmun N‐test) and 70% (IPA N‐test). On the other hand, the specificity of these tests was high. Of 1029 samples taken before an indication of infection, 97.4% showed a negative Euroimmun‐N‐test result and 84.5% showed a negative IPA N‐test result (Table 2b). In addition, in this group, 12 positive samples in the Euroimmun‐N‐test also showed an increase in antibodies against the S1 protein. This is a strong indication that there was a previous infection that was not detected by a PCR or antigen test. If these N‐test results are not evaluated as false positives, the specificity increases to 98.5% (Euroimmun‐N‐test) and 85.1% (IPA N‐test, Table 2c).

**Table 1:** Participants and vaccination of the study group.

	Participants [*n*]	Male [*n*]	Female [*n*]	Mean age [years]	Age range [years]
	126	43	83	47.7	18−67
Vaccination					
1 dose	2	2	0	45.0	35−55
2 doses	8	4	4	42.4	26−52
3 doses	54	17	37	45.3	20−64
4 doses	51	18	33	50.1	18−67
5 doses	11	2	5	52.7	32−62
Homolog	55	17	38	47.9	18−66
Heterolog	71	26	45	47.4	18−67
Vaccine (at least once)					
BNT162b2	123	41	82	48.0	18−67
AZD1222	60	20	40	47.3	18−64
mRNA‐1273	17	8	9	47.4	35−67

**Table 2:** Concordance of IgG IPA and Euroimmun nucleocapsid test results.

(a) Results of samples taken after the first positive SARS‐CoV‐2 PCR or antigen test				
	EuroImmun N test result			
IPA N test result	Positive *n* (%)	Borderline *n* (%)	Negative *n* (%)	Sum *n* (%)
Positive *n* (%)	32 (49%)	0 (0%)	2 (3%)	34 (52%)
Borderline *n* (%)	9 (14%)	2 (3%)	1 (2%)	12 (18%)
Negative *n* (%)	10 (15%)	1 (2%)	8 (12%)	19 (29%)
Sum *n* (%)	51 (78%)	3 (5%)	11 (17%)	65 (100%)
Only samples received 10−100 days after the positive PCR/antigen test are included. The percentages of positives indicate the sensitivity of the tests.				

(b) Results of samples from participants without infection or taken before infection				
	EuroImmun IgG N test result			
IPA IgG N test result	Positive *n* (%)	Borderline *n* (%)	Negative *n* (%)	Sum *n* (%)
Positive *n* (%)	7 (0.7%)	0 (0%)	34 (3.3%)	41 (4.0%)
Borderline *n* (%)	1 (0.1%)	0 (0%)	118 (11.5%)	119 (11.6%)
Negative *n* (%)	12 (1.2%)	7 (0.7%)	850 (82.6%)	869 (84.5%)
Sum *n* (%)	20 (1.9%)	7 (0.7%)	1002 (97.4%)	1029 (100%)
Only samples taken before a reported infection by questionnaire are included. The percentages of negatives indicate the specificity of tests.				

(c) Same as (b), but 12 samples with positive Euroimmun N test results and an increase of IgG to S1 were excluded (unreported)				
	EuroImmun N test result			
IPA N test result	Positive *n* (%)	Borderline *n* (%)	Negative *n* (%)	Sum *n* (%)
Positive *n* (%)	0	0	34 (3.3%)	34 (3.3%)
Borderline *n* (%)	0	0	118 (11.6%)	118 (11.6%)
Negative *n* (%)	8 (0.8%)	7 (0.7%)	850 (83.6%)	865 (85.1%)
Sum *n* (%)	8 (0.8%)	7 (0.7%)	1002 (98.5%)	1017 (100%)
The percentages of negatives indicate the corrected specificity of tests.				

### 3.2 Incidence of SARS‐CoV‐2 Infection

In the study population of 126 vaccinated participants, SARS‐CoV‐2 infection was reported in 82 participants. However, not all participants took part until the end of the study but dropped out at different times (Table 3). It is therefore possible that infections were not recorded, especially after early drop‐out. On average, evidence of SARS‐CoV‐2 infection occurred 163 days after vaccination but was observed as early as 16 days after the third vaccination and 17 days after the fourth vaccination. Of the 82 participants with evidence of SARS‐CoV‐2 infection, 53 reported symptoms, 4 had no symptoms, and 7 were identified only by IgG antibodies. No serious illnesses were reported. Symptom questionnaires were not available for 18 participants.

**Table 3:** Duration of participation until exit and/or infection with SARS‐CoV‐2.

	Participants [*n*]	Gap infection or exit after vaccination [days]			Duration of participation after 1 vaccination [days]		
		Minimum	Mean	Maximum	Minimum	Mean	Maximum
**SARS‐COV‐2 positive** a	**82**	**16**	**163**	**708**	**223**	**708**	**1027**
Positive after 2 vaccinations	3	164	179	193	344	542	747
Positive after 3 vaccinations	57	16	168	708	223	698	1015
Positive after 4 vaccinations	22	17	149	383	438	758	1027
**SARS‐COV‐2 negative**	**44**	**3**	**181**	**499**	**16**	**511**	**1025**
Exit after 1 vaccination	2	16	36	56	16	36	56
Exit after 2 vaccinations	8	3	70	178	66	131	241
Exit after 3 vaccinations	16	14	267	499	205	500	740
Exit after 4 vaccinations	13	25	165	376	449	721	986
Exit after 5 vaccinations	5	32	183	383	718	794	1025
**All**	**126**	**3**	**171**	**708**	**16**	**639**	**1027**

a 75 participants with positive SARS‐CoV‐2 PCR and/or antigen test, 7 participants with positive Euroimmun N test and S1 increase.

### 3.3 Antibody Levels Over the Course of the Study

**Figure 1:**
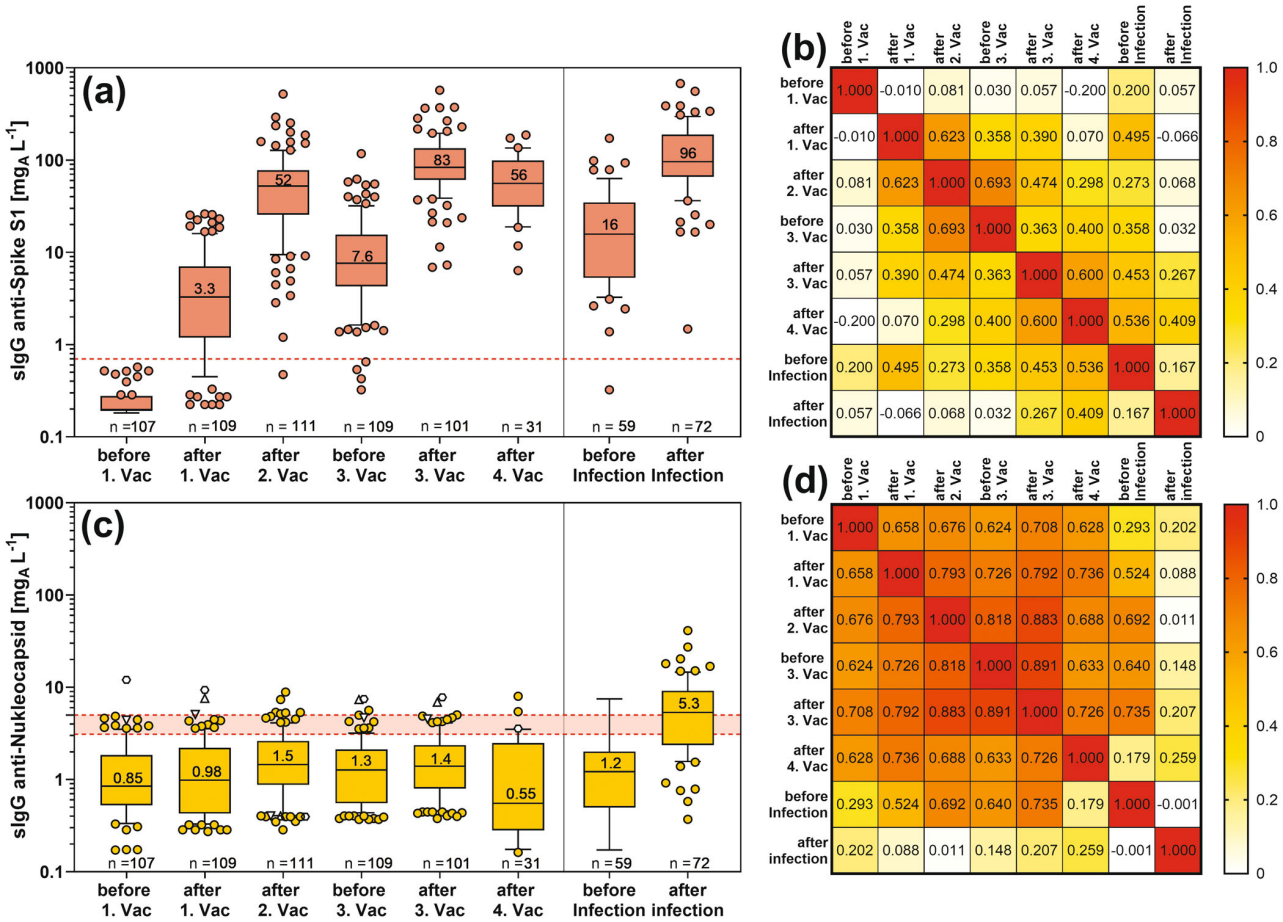
Box plots (a, c) and heatmaps of Spearman's correlations (b, d) of specific IgG concentrations to (a, b) the Spike‐S1 or (c, d) to the Nucleocapsid antigen from participants' sera before/after vaccination or infection. In the box plots (25%−75%, whiskers 10%−90%, median with value), the red dotted line marks the cut‐off levels for positive results (a), whereas the red shaded area marks borderline values (c). The same unfilled symbols in (c) belong to the same participants.

While 18% (20 of 109) had no IgG to the S1 protein after the first vaccination, this occurred in only one case after the second vaccination and no longer after the third and fourth vaccinations (Figure 1a). After each of the first three vaccinations, IgG concentrations increased significantly. The highest values were obtained after infection (median 96 mg_A_ L^−1^), although the values were not significantly higher than after the third or fourth vaccination (Kruskal−Wallis, Dunn's multiple comparison test).

Anti‐S1‐IgG levels were significantly correlated between samples after the first, second, and third vaccinations and between the third vaccination and after infection (Figure 1b). The ranks before the first vaccination were not significantly correlated with the ranks of the later samples, the ranks after infection were only significantly correlated with the ranks after the third vaccination, and the ranks after the fourth vaccination were only significantly correlated with the ranks before and after the third vaccination. The other ranks between the 1st, 2nd, and 3rd vaccinations were significantly correlated.

Anti‐nucleocapsid‐IgG antibodies remained at a low level over the course of repeated vaccinations (Figure 1c). After infection, IgG levels were significantly higher (median 5.3 mg_A_ L^−1^) than at any other time point. Some individuals had slightly elevated anti‐nucleocapsid‐IgG at baseline, which remained at similar levels throughout the study. This is consistent with the highly significant Spearman rank correlations between all pre‐infection samples (Figure 1d). All correlations were significant except for the postinfection ranks.

### 3.4 Comparison of IgG Results With Different Spike‐S1 Variants

In Germany, the first cases of SARS‐CoV‐2 infection caused by the Omicron variant (B.1.1.529) were reported in December 2021. By January 2022, Omicron had become the predominant variant in Germany, displacing the previously dominant Delta variant and accounting for the majority of infections from that point onwards [10]. The high transmissibility of the Omicron variant led to a sharp increase in the number of infected cases and was probably also the cause of the large number of infections in our study from 2022 onwards. We therefore developed an IgG test against the Omicron variant of the spike‐S1 protein (anti‐oS1‐IgG) and tested the sera taken immediately after vaccination as well as the sample before the third vaccination and all subsequent samples with this ELISA.

**Figure 2:**
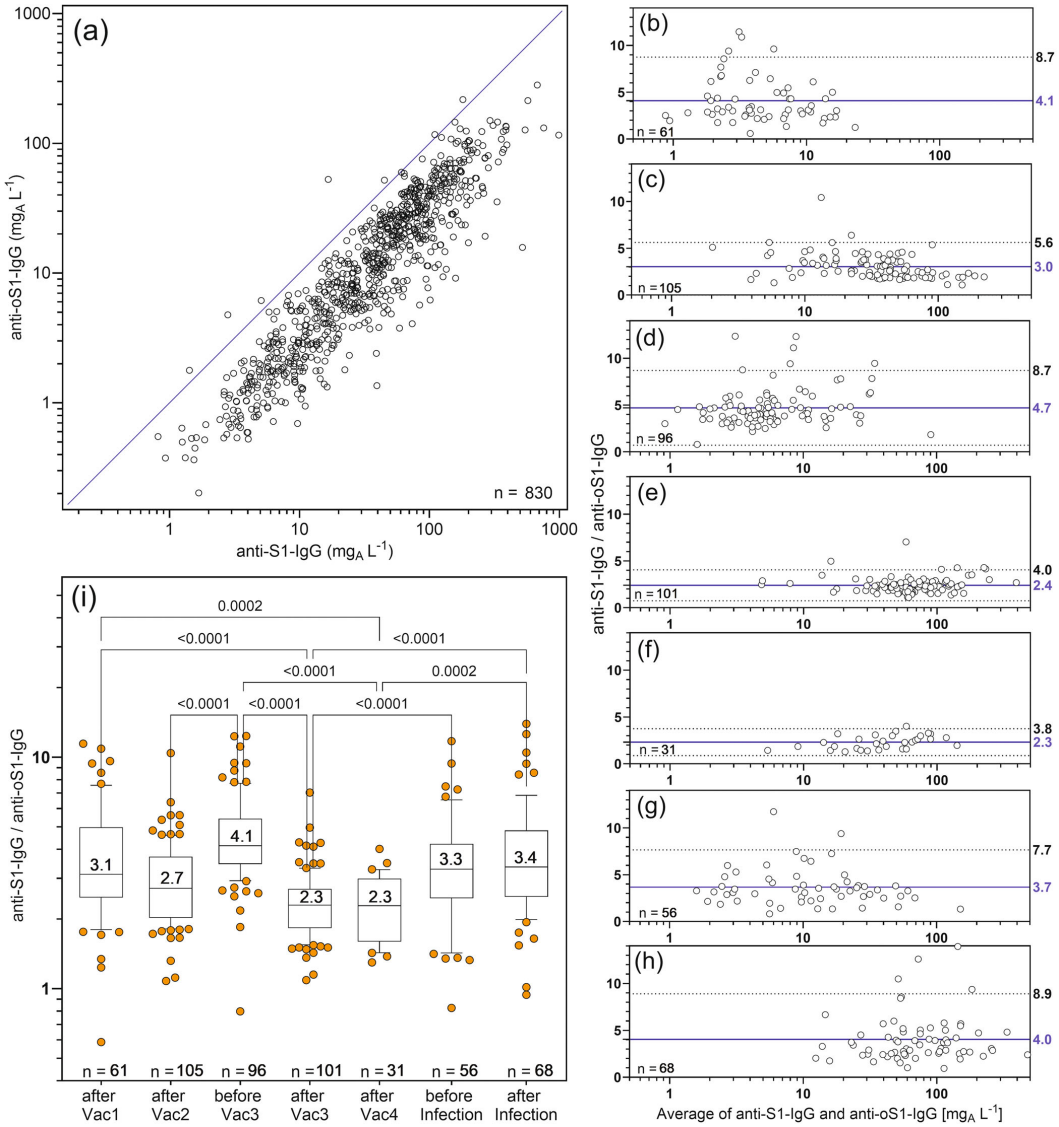
Comparison between IgG to the Wuhan‐type spike S1 protein (anti‐S1‐IgG) and IgG to the omicron variant of the spike S1 protein (anti‐oS1‐IgG). (a) Scatter plot of anti‐S1‐IgG versus anti‐oS1‐IgG with line of identity (only values above the detection limit of both assays are included). Bland−Altman plots of the ratios of IgG values to both S1 variants versus their average (b) after 1st vaccination, (c) after 2nd vaccination, (d) before 3rd vaccination, (e) after3rd vaccination, (f) after 4th vaccination, (g) before infection, and (h) after infection show mean values and 95% limits of agreement as lines and dotted lines, respectively. Only the values above detection limit of both assays and of the sample taken directly after or before the event are included. (i) Box plots (25−75, whiskers 10%−90%, median with values of the ratios of IgG values to S1‐variants). Significances were calculated with Kruskal−Wallis and uncorrected Dunn's test. Only significances with *p*‐values less than 0.001 were indicated.

In 830 samples, both the original IPA‐S1‐test and the IPA‐oS1‐test had values above the detection limits (Figure 2). The IgG values were highly significantly correlated (Spearman's *r* 0.918, confidence interval (CI) 0.906−0.928). Anti‐S1‐IgG was approximately three times (median 3.05; mean 3.75) higher than anti‐oS1‐IgG (Figure 2a). Analysis of the ratios (anti‐S1‐IgG/anti‐oS1‐IgG) before and after vaccination and infection revealed differences that were not dependent on the mean of IgG to both S1 variants in the Bland−Altman analysis (Figure 2b−h). Lower mean ratios and standard deviations were observed in samples taken shortly after vaccination 2 (Figure 2c), vaccination 3 (Figure 2e), and vaccination 4 (Figure 2f) than in samples taken after vaccination 1 (Figure 2b) or compared to the last sample taken before vaccination 3 (Figure 2d) and before (Figure 2g) or after infection (Figure 2h). The Kruskal−Wallis test showed that most of these differences were highly significant (Figure 2i).

### 3.5 Comparison of Spike and Nucleocapsid IgG Levels After Infection

Comparing the levels of IgG antibodies against the different antigens after infection, lower levels of antibodies were produced against the nucleocapsid (median 5.3 mg_A_ L^−1^, Figure 1c) than against the spike variants in the vaccinated group (median 96 mg_A_ L^−1^ against the Wuhan type, Figure 1a, 32 mg_A_ L^−1^ against the Omicron variant, data not shown). In contrast, levels of IgG directed against the nucleocapsid (150 mg_A_ L^−1^ anti‐N‐IgG) were significantly higher than those directed against the spike protein (40 mg_A_ L^−1^ anti‐S1‐IgG) when the SARS‐CoV‐2 WHO 20/136 reference, prepared from pooled plasma of convalescent donors with Covid‐19, was analyzed [6].

### 3.6 Half‐Life of sIgG After Vaccination or Infection

**Figure 3:**
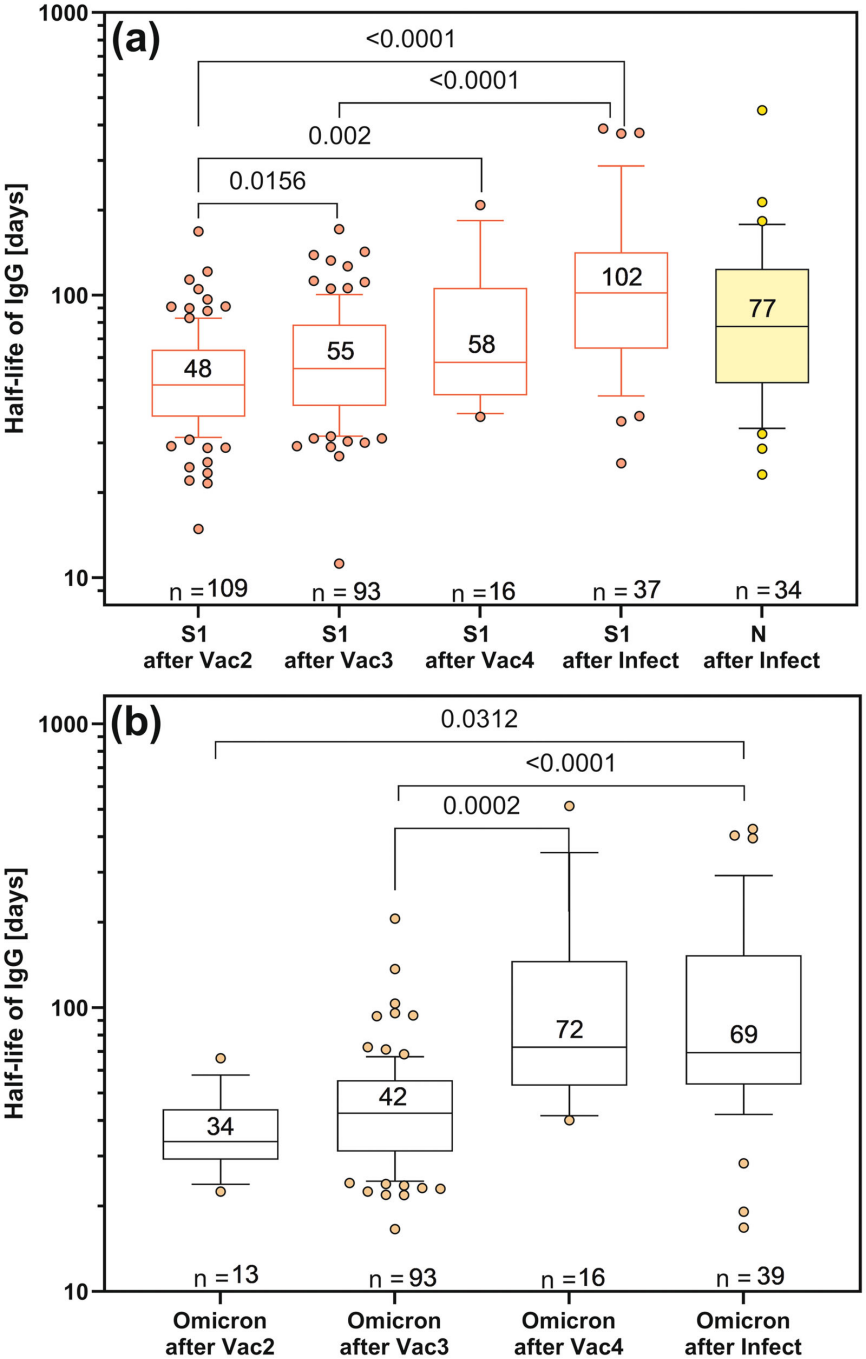
Box plots (25%−75%, whiskers 10%−90%, median with value) of half‐lives of IgG antibodies to (a) spike S1 and nucleocapsid N or (b) against spike omicron S1 after vaccinations and infection. Paired comparisons for the same participants at different study phases were calculated using the Wilcoxon matched‐pairs signed rank test with significances indicated by *p* values. Not all samples between vaccination 2 and 3 were measured in the anti‐oS1‐IgG assay, resulting in only 13 half‐life values.

The anti‐S1‐IgG and anti‐oS1‐IgG levels in samples taken after vaccination showed an exponential decrease as a function of time. If at least two samples were taken between vaccinations or after infections (there were only a few after the first vaccination and after the fifth vaccination), the decay constant and half‐life of specific IgG could be calculated (Figure 3). While the median half‐life of anti‐S1‐IgG was 48 days (95% CI: 44−56) after the 2nd vaccination, the median increased to 55 days (95% CI: 49−65) after the 3rd vaccination, and to 58 days (95% CI: 44−107) after the 4th vaccination. The highest half‐lives were observed after infection: anti‐S1‐IgG median 102 days, 95% CI: 75−135; anti‐N‐IgG median 77 days, 95% CI: 54−101 (Figure 3a). A similar increase in half‐life with further vaccinations was observed for anti‐oS1‐IgG (Figure 3b), particularly after the 4th vaccination, where the median half‐life was 72 days (95% Cl: 51−147).

The Wilcoxon matched‐pairs signed rank test was used to compare IgG antibody half‐lives in the same participants. Overall, the differences were significant (Figure 3). In addition, the pairing of half‐lives of IgG to S1 was significantly effective, except for ranks after vaccination 2 and ranks after infection (data not shown). In contrast, the pairing of IgG half‐lives to Omicron‐S1 was only significant between vaccinations 3 and 4 (Spearman's *r* = 0.6, *p* = 0.008).

When comparing the half‐lives of anti‐oS1‐IgG and anti‐S1‐IgG, the half‐lives of IgG to the Omicron variant after the second (median 34 days, 95% CI: 28−44) and third (median 42 days, 95% CI: 40−46) vaccinations were significantly lower than those of IgG to the original spike‐S1 after the second and third vaccinations, respectively (Wilcoxon matched‐pairs signed rank test, for vaccination 2 *p* = 0.0046, for vaccination 3 *p* < 0.0001), whereas the half‐life after the fourth vaccination was significantly higher for anti‐oS1‐IgG (*p* = 0.0353).

### 3.7 Influence of Vaccine, Gender, and Age

The specific IgG and half‐life rankings after vaccination and infection did not show significant differences (Mann−Whitney *U* test) between male and female participants. However, the vaccines used had an effect on anti‐S1‐IgG levels and, in some cases, on their half‐life. Anti‐S1‐IgG after the 1st and 4th vaccinations and after infection was significantly higher (Mann−Whitney) when only mRNA vaccines (BioNTec, Moderna) were used than when the AstraZeneca vaccine was used for the 1st vaccination dose (data not shown). In contrast, the half‐life of anti‐S1‐IgG after the second vaccination was significantly longer with heterologous vaccination using the AstraZeneca vaccine than with homologous vaccination using mRNA vaccines (Mann−Whitney *p* = 0.0086, median 56.5 vs. 44 days).

**Figure 4:**
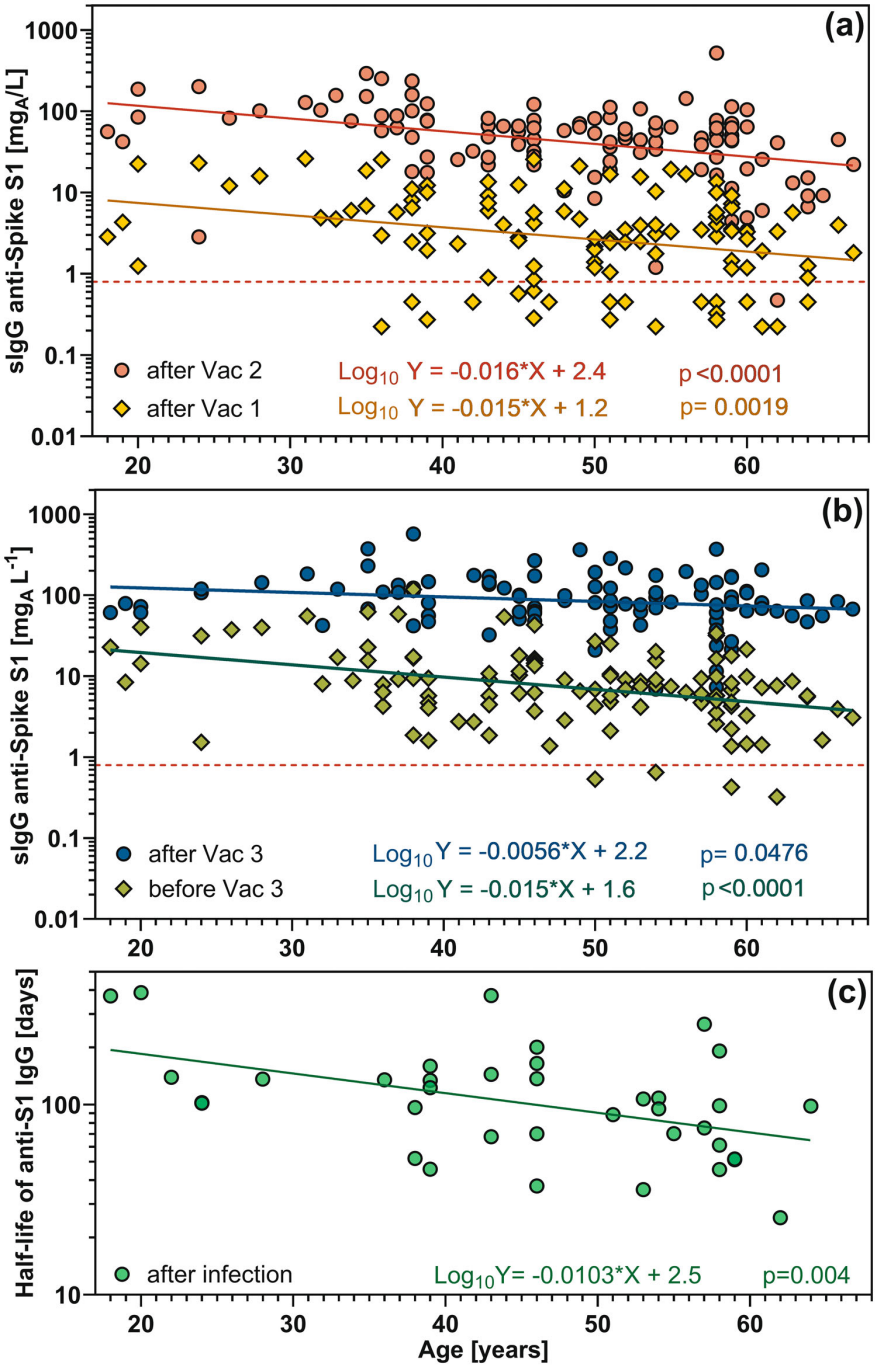
IgG levels to the spike S1 protein in relation to the age after (a) first and second vaccination, (b) before and after third vaccination, and (c) half‐life of IgG to the spike S1 protein after infection. The linear trend lines of the logarithmic values and the underlying functions are color‐coded. The slopes are significantly different from zero.

The age of the participants had a moderate effect on IgG to the S1 protein after the 1st, 2nd, before, and after the 3rd vaccination (Figure 4a,b). The slopes of the interpolated lines differed significantly from zero and were negative indicating an age‐related decrease in IgG concentrations. After the fourth vaccination and before and after infection, the slope of the age‐dependent trend line was not significantly different from zero (data not shown). However, when the age dependence of the half‐life of IgG to S1 after vaccination or infection was analyzed, only the values after an infection showed a significant influence of age (Figure 4c). The half‐life of IgG to S1 decreased with increasing age. However, no significant trend with age was observed for the half‐life of IgG to Omicron‐S1 or IgG to nucleocapsid after infection (data not shown).

## 4 Discussion

Our longitudinal prospective study began after SARS‐CoV‐2 vaccines became available in Germany and followed antibody levels in a small cohort of adult workers against two variants (Wuhan type and Omicron) of the spike S1 protein and the nucleocapsid proteins over 3 years after initial vaccination. A unique feature of the antibody quantification method that we developed before this study is that the levels can be traced back to international standards for immunoglobulin G, allowing the levels to the different SARS‐CoV‐2 antigens to be compared [6]. It should be noted that a quantitative ELISA using a reference of known IgG concentration measures the antibody binding capacity, which is influenced by both the amount of IgG and the avidity and affinity of the antibodies used. We therefore use the unit mg_A_/L for our IgG measurements.

While numerous commercial assays can quantify the level of IgG to the Wuhan type spike protein or its receptor‐binding domain (RBD) [11, 12, 13], usually relative to the international SARS‐CoV‐2 WHO 20/136 standard (pooled plasma from unvaccinated COVID‐19 recovered donors [9, 14]), there are mostly only qualitative assays for antibodies to the nucleocapsid.

When comparing the levels of IgG antibodies to the different antigens after infection, it is striking that far fewer antibodies appeared to be produced against the nucleocapsid (median 5.3 mg_A_ L^−1^, Figure 1c) than against the spike variants in the vaccinated group (median 96 mg_A_ L^−1^). This is consistent with the observation by many investigators that antibody concentrations against the spike protein rise sharply with repeated vaccination, often to levels higher than after mild or moderate COVID‐19 disease [15, 16, 17]. In contrast, median IgG‐concentrations against the nucleocapsid (13 mg_A_ L^−1^) were higher than those against the spike protein (4 mg_A_ L^−1^) in the unvaccinated COVID‐19 population studied during the development of our immunoassays. Similarly, the measured IgG concentration against the nucleocapsid (150 mg_A_ L^−1^ anti‐N‐IgG) was significantly higher than that against the spike protein (40 mg_A_ L^−1^ anti‐S1‐IgG) when the SARS‐CoV‐2 WHO reference preparation derived from unvaccinated COVID‐19 diseased donors was analyzed in our quantitative ELISAs. This difference in the quantitative ratio of IgG antibodies to both antigens between vaccinated and unvaccinated SARS‐CoV‐2‐infected individuals is consistent with milder symptoms in the vaccinated population. It is possible that the immune defense of the SARS‐CoV‐2 virus by the anti‐spike antibodies also minimizes the duration and intensity of contact with the nucleocapsid, so that fewer anti‐N antibodies are produced in vaccinated individuals. One consequence of the lower levels of anti‐N antibodies in vaccinated individuals is that the sensitivity of the anti‐nucleocapsid IgG test to detect SARS‐CoV‐2 infection is reduced compared to testing in unvaccinated populations. This is true not only for the quantitative anti‐N‐IPA test we developed, but also for the Euroimmun‐N‐test used in our studies as an adjunct test to detect infection. While the sensitivities of the anti‐N‐IgG tests in the unvaccinated group of infected individuals were estimated to be 89.6% (Euroimmun‐N‐test) and 83.3% (IPA‐N test) [6], the sensitivities in the vaccinated group fell to 83% (Euroimmun‐N‐test) and 70% (IPA‐N‐test). The reduced sensitivity of nucleocapsid antibody tests has implications for seroprevalence and thus estimates of SARS‐CoV‐2 infection in a population. It is possible that these rates, already considered high, are still underestimated.

In our study population of repeatedly vaccinated employees, the infection rate was also high, with at least 82 out of 126 participants (65%) identified as infected, mostly by reported positive PCR test results. The vast majority of infections occurred at a time when the Omicron variant was prevalent, and even triple vaccination did not provide adequate protection against infection in all cases. This variant of the spike‐S1 protein differs from the Wuhan type of the virus by 30 mutations, which also results in reduced recognition by postvaccination IgG antibodies. Our quantitative IgG tests show an average reduction in recognition of the Omicron‐S1 protein by a factor of three compared with the Wuhan type used in vaccinations. This relationship was not constant over the course of the longitudinal study. Immediately after repeated vaccination, the differences and the variation of these differences were significantly smaller, whereas after the first vaccination, the differences and variation were larger and increased at longer intervals after vaccination and also after infection. These differences between the IgG levels to Omicron and the Wuhan variant of the spike‐S1 protein are clearly visible in the Bland−Altman plots. While the ratio of anti‐S1‐IgG to anti‐oS1‐IgG is 3.8−5.6 at the upper 95% limit of agreement shortly after revaccination, it reaches values of 7.7−8.9 at the other time points. This suggests that the half‐life of anti‐oS1 IgG is shorter than that of anti‐S1 IgG and that the latter also varies more between individuals. At least for the half‐lives after the second and third vaccinations, this can be confirmed in our collective: While anti‐S1‐IgG had median half‐lives of 48 and 55 days, the half‐lives for anti‐oS1‐IgG were 34 and 42 days after the second and third vaccinations, respectively. Interestingly, however, the half‐life of anti‐oS1‐IgG after the 4th vaccination was significantly longer than that of anti‐S1‐IgG. This may be due to the fact that in the majority of cases, the vaccine used for the 4th vaccination contained mRNA based on sequences of the Omicron variant in addition to the Wuhan type variant. The increased half‐life of anti‐oS1‐IgG may indicate a later onset of antibody production against Omicron‐specific epitopes compared to production by pre‐existing B cells. However, the half‐life of anti‐oS1‐IgG was not increased after infection compared to anti‐S1‐IgG.

Overall, by determining concentrations and half‐lives with our quantitative immunoassays, we were able to determine an increase in both IgG levels and their half‐lives with each subsequent vaccination. An increase in antibody levels to variants of the spike‐S1 or the RBD with repeated vaccination has also been observed in other studies using other immunoassays [15, 18, 19]. In addition, a drop in IgG levels was often observed with time after vaccination [12, 19, 20], although the half‐life was rarely determined. De Boer et al. had determined half‐lives using a multiple dilution method [21]. For anti‐S1‐IgG, they determined a median half‐life of 127 days in a naturally infected group compared to 53 days after a twofold vaccination [21]. The latter is not very different from the half‐life of 48 days found in our study. Van Elslande et al. had compared the half‐life of anti‐S1 and anti‐N IgG in naturally infected individuals and found that the former had a half‐life of 199 days, more than twice as long as the latter's 76 days [22]. In our study, we found a similar half‐life for anti‐N IgG (77 days) in vaccinated individuals after infection. However, the observed difference with the anti‐S1 IgG half‐life in our vaccinated cohort was not as great as in van Elslande's study.

We also looked at what factors influenced antibody levels and half‐lives. While gender had no significant effect on our collective, there was a moderate effect due to the age of the participants. With increasing age, there was a significant trend towards lower levels of anti‐S1‐IgG, but only after the first three vaccinations, and a shorter half‐life of these antibodies after infection. However, the differences between individuals were much larger than the differences due to older age, which explains why significant differences were sometimes found when comparing age groups [12, 20] and why these differences were not significant in other studies [18]. As often reported [5, 15, 20, 23], we also found differences between the mRNA vaccines and the AstraZeneca vaccine in our cohort. Anti‐S1 antibodies were lower after the first vaccination and even lower after the fourth vaccination when the AstraZeneca vaccine was used for the first vaccination. However, the heterologous second vaccination showed a significantly longer half‐life than those vaccinated with mRNA vaccines alone.

Although our study had only a few participants, we analyzed whether higher antibody levels after vaccination were associated with better protection against infection, but found no significant differences [24]. Results from large studies suggest that higher anti‐S1 IgG levels do confer better protection against infection [25]. However, the protective effect is small and outweighed by other factors such as individual risk behavior.

Weaknesses of our study include the rather small number of people studied and the fact that data on SARS‐CoV‐2 infections were obtained only by questionnaire. In addition, the assay we developed only measures the binding capacity of anti‐S1 antibodies and is not limited to neutralizing antibodies. However, several studies have reported a very good correlation between anti‐S1 IgG and neutralizing antibodies [26, 27, 28, 29]. In addition, we have compared some of our anti‐S1 IgG results with a neutralization assay developed in Watzl's laboratory [30]. A total of 87 samples from individuals after the 2nd and 3rd BNT162b2 vaccinations were also measured in the neutralization assay. The Spearman's correlation compared to the results of anti‐S1‐IgG levels was high (data not shown).

A strength of our study is its prospective longitudinal design over a period of 3 years. Although our immunoassays may not provide identical results to commercial assays used in much larger studies, they do provide quantification through traceability to international IgG references, which allows some comparability of antibody binding capacity to different SARS‐CoV‐2 antigens and their changes over time.

## 5 Conclusions

Despite repeated vaccinations and the subsequent increase in IgG antibodies against the spike S1 protein, as measured by the quantitative immunoassays, the majority of the cohort reported breakthrough SARS‐CoV‐2 infections during the study period. This phenomenon may be partially explained by the approximately threefold reduction in IgG binding to the Omicron variant relative to the ancestral Wuhan strain of the spike S1 protein, in combination with the significantly shortened half‐life of these antibodies.

## Author Contributions

Monika Raulf, Ingrid Sander, and Sabine Kespohl conceived and designed the study. Monika Raulf and Thomas Brüning acquired the funding and administered the project. Alexandra Beine, Kerstin Belting, Jürgen Bünger, Simon Weidhaas, Ingolf Hosbach, and Christian Eisenhawer recruited and investigated the study cohort. Ingrid Sander, Monika Raulf, Jan Gleichenhagen, and Sabine Kespohl developed and supervised the laboratory methods and analysis. Philipp Göcke contributed resources. Ingrid Sander performed the formal analysis, visualization, data curation, and validation. Ingrid Sander wrote the original draft, which was reviewed, edited, and approved by all authors.

## Conflicts of Interest

M.R. received lecture fees from the following companies and associations between 2022 and 2025: AlkAbello Arzneimittel GmbH, Berufsverband Deutscher Baubiologen VDB e.V., LetiPharma, and ThermoFisher Scientific (Phadia). C.E. prepares expert reports on recognized occupational disease cases of a SARS‐CoV‐2 infection on behalf of the German Social Accident Insurance. P.G. is a shareholder in his laboratory association. The other authors declare no conflicts of interest.

## Data Availability

The data that support the findings of this study are available on request from the corresponding author. The data are not publicly available due to privacy or ethical restrictions.
